# Q-Meter: Quality Monitoring System for Telecommunication Services Based on Sentiment Analysis Using Deep Learning

**DOI:** 10.3390/s21051880

**Published:** 2021-03-08

**Authors:** Samuel Terra Vieira, Renata Lopes Rosa, Demóstenes Zegarra Rodríguez, Miguel Arjona Ramírez, Muhammad Saadi, Lunchakorn Wuttisittikulkij

**Affiliations:** 1 Department of Computer Science, Federal University of Lavras, Minas Gerais 37202-000, Brazil; samuel.vieira1@estudante.ufla.br (S.T.V.); demostenes.zegarra@ufla.br (D.Z.R.); 2 Department of Electronic Systems Engineering, University of São Paulo, São Paulo 05508-010, Brazil; maramire@usp.br; 3 Department of Electrical Engineering, University of Central Punjab, Lahore 54590, Pakistan; muhammadsaadi@gmail.com; 4 Wireless Communication Ecosystem Research Unit, Department of Electrical Engineering, Chulalongkorn University, Bangkok 10330, Thailand

**Keywords:** telecommunication services, online social network, sentiment analysis, QoE, sensing, deep learning

## Abstract

A quality monitoring system for telecommunication services is relevant for network operators because it can help to improve users’ quality-of-experience (QoE). In this context, this article proposes a quality monitoring system, named Q-Meter, whose main objective is to improve subscriber complaint detection about telecommunication services using online-social-networks (OSNs). The complaint is detected by sentiment analysis performed by a deep learning algorithm, and the subscriber’s geographical location is extracted to evaluate the signal strength. The regions in which users posted a complaint in OSN are analyzed using a freeware application, which uses the radio base station (RBS) information provided by an open database. Experimental results demonstrated that sentiment analysis based on a convolutional neural network (CNN) and a bidirectional long short-term memory (BLSTM)-recurrent neural network (RNN) with the soft-root-sign (SRS) activation function presented a precision of 97% for weak signal topic classification. Additionally, the results showed that 78.3% of the total number of complaints are related to weak coverage, and 92% of these regions were proved that have coverage problems considering a specific cellular operator. Moreover, a Q-Meter is low cost and easy to integrate into current and next-generation cellular networks, and it will be useful in sensing and monitoring tasks.

## 1. Introduction

Online social networks (OSNs) allow access to information about different topics, and users share their positive or negative opinions according to their experiences [1]. Thus, measuring the sentiment polarity of written sentences in OSNs can help to discover user satisfaction or dissatisfaction with a particular object or service.

Sentiment analysis can be applied in several areas [2,3,4], such as the discovery and monitoring of failures of a service [5] or product and the performance improvement of recommendation systems [6]. However, sentiment analysis tools are very specific, focusing on determined topics, which do not include communication system problems. Thus, sentiment analysis can be useful for sensing the users’ opinion about diverse services, such as telecommunication services in next generation 5G or 6G-based sensor networks, improving the users’ quality of experience (QoE).

Sentiment prediction of sentences posted on OSN [7] can be performed by machine learning (ML) algorithms. The ML algorithms can be used in several research areas [8,9,10,11,12], obtaining high accuracy in review datasets using a recursive neural tensor network (RNTN), according to [13]. High accuracy is reached when the sentiment and emotions are determined from images and speech signals [14]. However, the sentiment analysis applied to texts is more complicated to perform [15]. The results for positive and negative sentiments can reach an average precision, recall, and F-measure of 73.77%, 74.01% and 73.80%, respectively, in some works [16]. The emotion classification accuracy was not superior to 80% in [17]. Another work [18] performed a textual sentiment analysis by three different attention convolutional neural networks (ACNNs) and cross-modality consistent regression, and the accuracy results were not higher than 88%. Irsoy and Cardie [19] presented a deep recursive neural network (DRNN) with fine-grained sentiment prediction, and the accuracy of binary sentiment classification was 86.6%. Convolutional neural networks (CNNs) and bidirectional long short-term memory (BLSTM)-recurrent neural networks (RNNs) have been used for measuring sentiment analysis [20,21]. However, the context of the studies is not communication services.

Because of the good performance of the CNN with BLSTM-RNN in other areas [22,23], this algorithm was chosen to be used in this work for performing sentiment analysis in the area of telecommunication (telecom) services.

A deep neural network can have good performance depending on the activation function that is used. Thus, the activation function is an important step in neural networks, providing a nonlinear property for this kind of networks [24]. In recent years, many activation functions have been proposed to replace known functions, such as rectified linear units (ReLUs) [25], including randomized leaky ReLUs (RReLUs), Swish [26], Maxout [27] and others [28]. soft-root-sign (SRS) [29] activation function, which has a better generalization performance as well as faster learning speed, can adaptively adjust the output through a pair of independent trainable parameters. Thus, the SRS was chosen to be implemented in this work because of the learning speed.

Currently, there are different studies about quality monitoring in cellular communication networks [30,31,32,33]. The quality can be measured by the quantity and location of RBS [34] that are operating in a specific region. In [35], a model is proposed that classifies churn customer data using classification algorithms. The random forest (RF) algorithm performs 88.63% of correctly classified instances. However, sentiment analysis is not approached in [35], and no monitoring system is proposed for telecom services.

It is important to note that the signal strength at the user locations can be measured by the network operator. However, in many situations in which the user is inside of buildings, the signal strength may vary and these data are difficult to be determined by the operator. Thus, the OSN, provided with a quality monitoring tool, can warn when there are failures not reported in the data collected by the operators.

The goal of a monitoring system related to telecom services is to show network operators why a call has an unsatisfactory quality. Thus, the main objective of this work is to improve user complaint detection for telecom services through a deep learning algorithm, the CNN with BLSTM-RNN and SRS. This technique presents a high potential to classify important topics with a fast learning speed. Thus, the telecom industry will benefit from the proposed Q-meter, measuring the communication quality in a specific region, easily extracting data from OSN. The sentences containing the name of the main cellular operators in Brazil and respective geographical location are extracted from an OSN. The sentences are sent to the CNN with the LSTM-RNN algorithm for sentiment analysis and topic classification. The map of signal propagation over a determined region is verified and sent to the network operator. The number of RBS for a specific geographical area and other quality indicators are obtained from an open Brazilian database from the National Telecommunications Agency (ANATEL) to validate the quality of a call in a specific region. The coverage of the cellular signal is also verified using a developed application containing a terrestrial RF path. Thus, the monitoring system proposed in our work, named the Q-Meter, is built.

In this work, other sentiment metrics are used for comparison to the CNN with BLSTM-RNN and SRS results, such as the SentiMeter-Br [2] and LSTM with a gated recurrent unit neural network (GRU) model [36]. All these metrics are trained on the telecom scenario in the Brazilian Portuguese language.

The main contributions presented in this paper are summarized as follows:

1. A method to classify the main subscriber complaints about cellular operator services based on OSN information is proposed. This work focuses on the weak signal problem because it was the most recurrent users’ claim. However, other topics can be easily addressed.
2. The convolutional neural network (CNN), a bidirectional long short-term memory (BLSTM), recurrent neural network (RNN), and the soft-root-sign (SRS) activation function are tested and validated as a sentiment analysis tool in the area of telecommunication services. The proposed model overcame other similar machine learning algorithms.
3. Specific regions with possible coverage problems are automatically detected through the OSN. Thus, the Q-Meter can work as a real-time quality monitoring system for mobile communication networks.
4. In general, the proposed Q-Meter will be useful for network operation and maintenance tasks, and its integration with commercial cellular networks, such as current and next generation 6G networks, will help to improve the network performance, and therefore, the users’ QoE. It is important to note that the Q-meter should not require either significant investments or complicated engineering operations.

The results show that the CNN with BLSTM-RNN and SRS reaches a precision of 0.98 for negative sentence classification. Other machine learning algorithms are tested for comparison to the deep learning solution, and the CNN with BLSTM-RNN presents better accuracy results. It is important to note that there are few works about SRS implementation and tests in CNNs with the BLSTM-RNN algorithm.

The remainder of this paper is organized as follows. Section 2 presents the related work and main concept definitions. Section 3 presents the proposed monitoring system. Section 4 presents the experimental results and discussions. Finally, Section 5 presents the conclusions of this article.

## 2. Related Work

In this section, the major works on sentiment analysis and monitoring systems for telecom services are discussed.

### 2.1. Sentiment Analysis

Currently, OSNs are used for data collection in many areas [37,38,39,40]. Users access the OSN to create a profile to share photographs, specific or general information, and join groups of friends [41,42]. The OSN is also used for emotion sharing, in which users’ emotional needs are reported and expressed [43,44,45].

Service satisfaction can also be measured in the OSN [46]. The sectors vary, such as banking companies [47,48,49,50], insurance companies [51,51], Internet services [52], the telecom industry [53,54,55] and others [56]. An overview is reported about classification techniques used for service satisfaction in the telecom area in [55], and it analyzes a benchmark of the used techniques. Studies [57] show that some metadata related to the time execution of the digital actions and patterns help to analyze digital work behaviors considering the distribution of such behaviors during a day or week.

The OSN provides a large quantity of data, which must be filtered for appropriate use [58]. The process of data mining is not always an easy task [59], and sentiment analysis is one of the tools that helps in the filtering task [60] in which sentences can be evaluated as having positive or negative polarity [61]. Sentiment analysis is considered a common practice for measuring the level of user satisfaction or dissatisfaction since its origin [62]. Through the dictionary of words or lexical analysis, a list of words, such as WordNet [62] and SentiMeter-Br [2], are formed to score the user sentiments. However, many of the existing dictionaries were developed to be used in general topics or they need to be periodically updated with new slang, such as in [2,6,63,64,65]. Thus, some works use the machine learning approach [66,67,68], which can be updated automatically.

The SVM algorithm is commonly used for emotion classification [69,70], presenting good generalization properties [71]. The RF and NB algorithms are also used for sentiment analysis with text classification in social networks [72].

Artificial neural networks are also used to recognize patterns in sequences of data, such as the RNN. The long short-term memory (LSTM) units are units of an RNN. The deep learning approach has been explored in various areas in recent years, such as image [73] and speech recognition [74]. Recently, deep learning has also been applied to text classification, and it has obtained accuracy higher than other machine learning algorithms for classifying text models [13]. Recent studies on CNNs presented significant performance improvements in natural language processing (NLP) tasks [75]. The Bi-LSTM outperforms other neural network models, which means that the deep Bi-LSTM can deal with the recognition of the state of other variants of RNN [76]. However, there are scarce works related to the use of deep learning for sentiment analysis applied in telecom services. CNN model was applied for churn prediction in [77]. However, the study was limited to classifying customers into churners and nonchurners. Almuqren et al. [36] studied Saudi telecom companies using sentiment analysis for customer satisfaction based on a corpus of Arabic tweets. The LSTM and GRU were used in [36], and 95.16% accuracy was obtained. The result in [36] found the polarity of tweets as being positive or negative. However, the study [36] identified general topics, such as ’have problems’ or ’greetings’ and not specific topics, such as ’weak signals’, in telecom services.

In deep learning algorithms, the activation function has an important role. A popular and widely used activation function for neural networks is ReLU, which was first proposed for RBM and afterwards for other kinds of neural networks. In recent years, various other activation functions have been proposed to replace ReLU. Leaky ReLU (LReLU) [78], replaced the negative part of ReLU with a linear function with superior results. Other variants have also been studied [26], but they present problems in gradient-based optimization. Compared with ReLU and variants, LReLU and others, ELU provides fast convergence and a clear saturation plateau in its negative region, with the advantages of learning more features. Based on these facts, the variants of ELU also demonstrated similar performance improvements [79]. However, the incompatibility between these activation functions and the problem of batch normalization (BN) has not been treated in depth. The Swish [26] function has been studied as an optimization method for search algorithms and reinforcement learning. However, the nonlinearity is very dependent on the network architecture. The activation function SRS [29], designed to solve potential disadvantages of ReLU, has shown smooth, nonmonotonic, and bounded characteristics, presenting faster learning and better generalization performance. Additionally, SRS improves its compatibility with BN, and it reduces the sensitivity to initialization. However, the SRS activation function has not been tested in a CNN with the BLSTM-RNN algorithm.

In this work, the CNN with the BLSTM-RNN algorithm is compared to the other metrics, such as the SentiMeter-Br and the LSTM with the GRU model used in [36].

### 2.2. Monitoring System for Telecommunication Services

Although there is concern about mobile communication availability, data collection work on telecom services is still scarce [80,81]. In [80], the authors focused on the study of the potential of extracting a large quantity of data, especially on telecom operators, to identify the most recurring use cases. Ref [80] studied how to reduce the rate of evasion or churn rate, increasing the average revenue per user (ARPU) and geographic marketing of companies. However, the study [80] addressed only the potential of data extraction by operators. In [81], the authors treated the integration of big data collected from the company with network optimization to improve the users’ quality experience. In [81], a framework for mobile network optimization was proposed, and the study presented the characteristics of data that are collected not only from the user’s equipment but also from mobile networks.

Other studies focus on churn prediction about telecom services [47,77,82,83,84]. However, they do not correlate the OSN data with global positioning system (GPS) data and signal propagation data. A hybrid model-based learning system was used in [82], which integrates supervised and unsupervised techniques for predicting customer behavior. The system combines a modified k-means clustering algorithm and a classic rule inductive technique. In [47], a churn prediction framework was performed to generate training data from customer records and leverage it for predicting customer churn within multiple horizons using standard classifiers. Some studies [83] show that deep learning models perform equally as well as traditional classifiers such as SVM and RF, selecting the better customer attributes for churn prediction. The neural network-based methodology for customer prediction in the telecom sector was studied in [85]. Studies [86] provided a comparison of customer disclaim prediction using artificial neural networks and decision trees, in which customer loyalty is measured in prepaid mobile phone organizations. Other studies [87] on predictive models for customer characteristics regarding mobile phone companies were performed, in which many classification algorithms were tested, such as Naive Bayes (NB), K-nearest neighbor (KNN), gradient boosted tree (GBT), single-rule induction (SRI) and deep learner neural net (DP), for customer characteristic prediction. In [87], the model based on NB outperformed the transformed data, and the DP, KNN and GBT algorithms performed on average.

In contrast to the studies already cited, in this work, the data from users are collected from the Twitter OSN. Additionally, the SRS activation function is tested in the CNN with the BLSTM-RNN algorithm. Such data can be captured and made available to any user and operators without access restrictions and are used in the proposed telecom measurement and monitoring system, the Q-Meter.

## 3. Methodology

This section presents the methodology of the proposed system named Q-Meter, the subjective tests for performing topic classification and sentiment metric evaluation, and the performance metrics used in this work.

### 3.1. Proposed Measurement System Monitoring Telecommunication Services, Q-Meter

**Figure 1:**
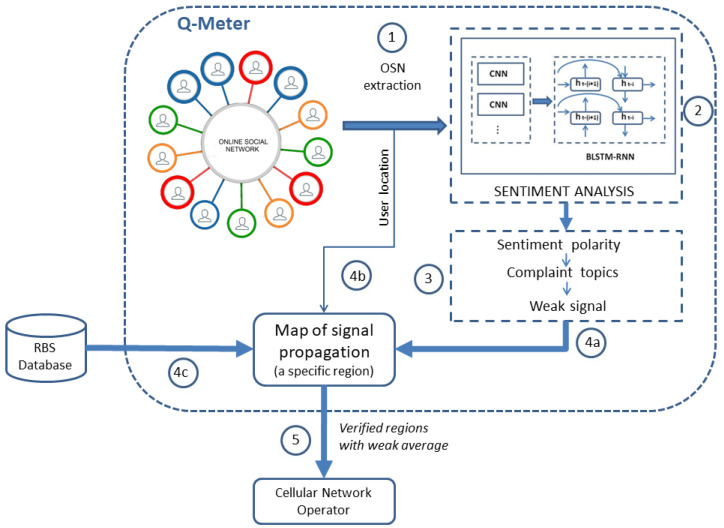
Steps for performing the proposed system named Q-Meter.

Figure 1 shows the steps for performing the measurement by proposed Q-Meter system. Sentence (1) is extracted from the OSN, and the data are used to feed the sentiment analysis algorithm (2). The sentiment analysis is performed by a deep learning model. The output of (2) is the sentiment polarity classification into positive or negative and the complaint topic classification (3) into weak signals or other topics. The weak signal complaint (4a) is sent to a map of signal propagation, which is performed by software. It is important to note that only the weak signal complaint is used in this work. However, any other complaint can be used. The weak signal complaint (4a) and the user location (4b) of the owner of the sentence are searched in radio propagation modeling software, and the RBSs are searched in a software database (4c). The output of Figure 1 is verified regions with weak averages, in which information is sent to a cellular network operator (5).

#### 3.1.1. Online Social Network (OSN) Extraction

The Twitter OSN was selected for data extraction because it is public and has a considerable amount of available information [88].

The REpresentational State Transfer (REST) application programming interface (API) [89] was used for data extraction, which was embedded in a PHP script for better visualization of the results. The extraction of the sentences was performed through keywords that represented possible complaints related to four main cellular operators in Brazil.

In total, 70,140 sentences were collected from Twitter, whose keywords were “problem”, “bad”, “complaint”, “horrible”, and “dissatisfied”, among others that represented user dissatisfaction, followed by the name of the mobile operator. Thus, each operator had approximately 17,535 tweets related to complaints during the period of 1 month of data collection.

From the sentences collected, the greatest difficulty was the similarity of the name of some Brazilian telecom operators with words of common use in OSN. Thus, prefiltering was built in such names.

Most of today’s mobile devices have GPS applications [90]. Therefore, the geographic region of the user who writes a sentence on the OSN can be located at the exact moment of the post. Certain repositories of quality indicators of telecom services indicate the geographic region of an RBS [91], but they do not consider the user’s opinion on the displayed indicators of the repository. Some machine learning algorithms were tested with the aim of obtaining the best accuracy in sentiment analysis and category, and only the algorithms with the best accuracy are shown in this work.

#### 3.1.2. Sentiment Analysis

In a sentence extracted from Twitter, the user who reported dissatisfaction regarding the service offered by a cellular operator and the region has the location saved. The geographic region of the user who wrote the sentence is obtained in case the GPS is activated.

The network architecture for analyzing sentence meaning through character-level representations by using a combination of LSTM and BLSTM with CNN presents good results for lexical classification and labeling tasks according to studies [92,93]. To compare the BLSTM-RNN performance, other algorithms were used: NB, SVM, RF and MP.

This research used the Theano library [94] for the deep learning architecture and other algorithm implementations [95].

The classification of sentiment polarity was performed in a binary attribute as negative/positive sentences. Of the total classified sentences as positive and negative sentences and topics, 80% were used for training, and 20% were used in the validation phase.

In the deep learning architecture, the CNN is used to compute the character-level representation of the sentences, helping to identify the negative or positive polarity and the topics. The convolutional kernel of the CNN algorithm performs the convolutions for the characters of the words in the sentences. For each convolution *i*, the kernel output kt is computed, as shown in Equation (1).

(1) $$kt_{i}=tanh\left(Mt_{1}\right)r_{li}+c_{1}$$

where the parameter $Mt_{1}$ is the parameter matrix, $c_{1}$ represents the learned bias vector, tanh represents the hyperbolic tangent activation function and $r_{li}$ is the character-level representation of the word *i*.

The SRS activation function was chosen to be used in this work and is defined in (2).

(2) $$SRS\left(i\right)=\frac{i}{\frac{i}{\alpha }+e^{-\frac{i}{\beta }}}$$

where the α and β variables are a pair of trainable positive parameters. The SRS presents a nonmonotonic region in which i<0 provides the zero-mean property. When i>0, it avoids and rectifies the output distribution. The derivative of SRS is defined as in (3).

(3) $$SRS^{\prime }\left(i\right)=\frac{\left(1+\frac{i}{\beta }\right)e^{-\frac{i}{\beta }}}{{\left(\frac{i}{\alpha }+e^{-\frac{i}{\beta }}\right)}^{2}}$$

The SRS has bounded output with the range $\left[\frac{\alpha \beta }{\beta -\alpha e}\right],\left[\alpha \right]$.

In the experiments, other activation functions were used, such as softmax and ReLU, for comparison to the SRS.

In the neural network model, the output vectors of BLSTM are used as input in the tahn layer. The character-level representation serves to feed the BLSTM-RNNs. The output of BLSTM is sent to the polarity extraction (PE) layer to choose the label sequence.

**Figure 2:**
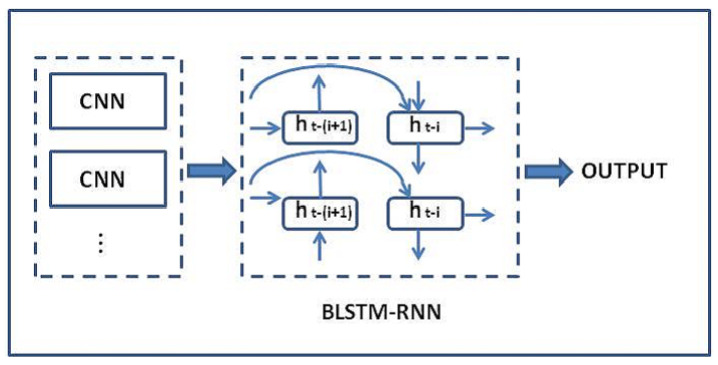
Topology of the CNN algorithm as input for the BLSTM-RNN algorithm, computing the hidden sequence *h*.

The hidden states, $h_{t-(i+1)}$ and $h_{t-1}$, capture information in direct and reverse directions. The LSTM output performs bottom-up and top-down computations for classifying the relation of the sentence. Figure 2 presents the topology of the CNN algorithm as input for the BLSTM-RNN algorithm, computing the hidden sequence *h*. OUTPUT represents the sentiment of the sentence. Only the negative sentences are selected and used as entrances to the map of signal propagation.

#### 3.1.3. Complaint Topics Classification

After sentiment polarity is performed on the extracted sentences, complaint topic classification is performed. The classification is separated into weak signals or other topics. Other negative complaints could be selected, such as poor customer service, many advertising messages, and call costs. However, this work focuses only on the weak signal complaint.

#### 3.1.4. Map of Signal Propagation

The Q-Meter considers only the sentences with negative sentiment polarity and the topic of weak signals. The location recorded by the GPS in the Twitter sentence is captured, and the data of this region are extracted from a global database available on the Internet. The location of the RBS is verified in an open Brazilian textual database to determine the veracity of complaints related to weak signals and the absence of RBS in a given region. The signal strength information is available for each region.

**Figure 3:**
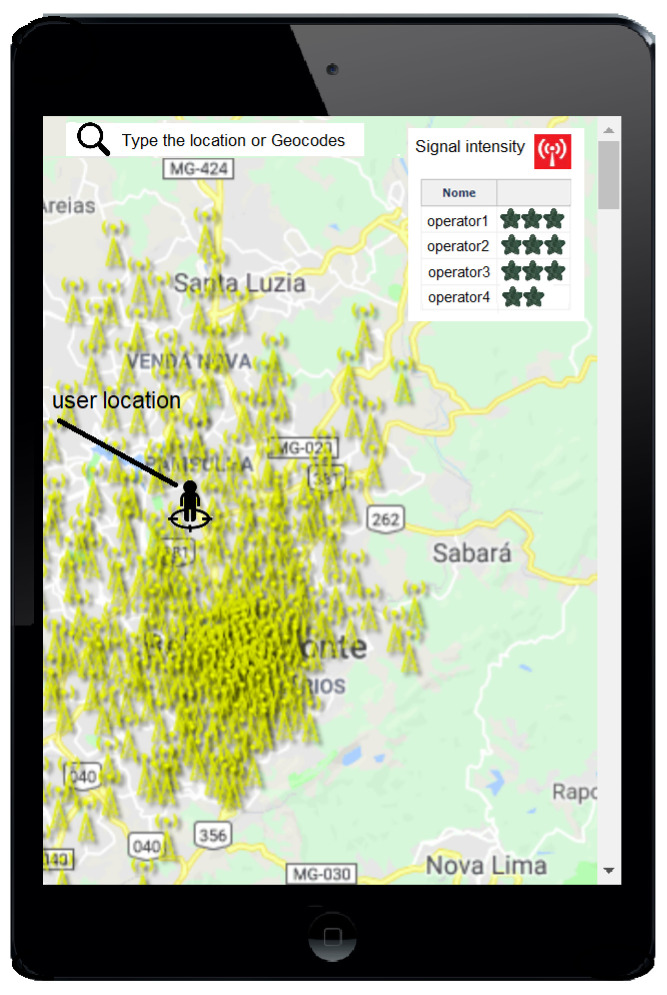
Cellular intensity of a region of Belo Horizonte, Minas Gerais, Brazil according to the Q-meter system.

Figure 3 shows the implemented map with the RBS of a determined region, in which RBS data were extracted from the textual open database. Through the map with radio propagation modeling, it is possible to verify the signal strength of the cellular operators in the region of Belo Horizonte, Minas Gerais (MG), Brazil. Three stars in Figure 3 represent a strong signal of a determined operator, and one star represents a weak signal.

After verifying the signal of the geographic region in which the user posts on Twitter, it is possible to validate or not validate the veracity of the information from the OSN.

The developed software is used to verify the comprehensiveness of the ERB signals.

#### 3.1.5. Cellular Network Operator

The weak signal regions and all the data about the complaints are sent to a cellular network operator. With this data, it is possible to apply mechanisms for improving user QoE.

### 3.2. Subjective Tests for Topic Classification and Sentiment Metric Evaluation

Subjective tests were performed in this work to evaluate the sentiment polarity and the topic classification performed by the deep learning model. Face-to-face evaluation was performed in a laboratory environment by Portuguese native-speaking assessors. There were 186 assessors to perform the tests, comprising 94 men and 92 women with ages ranging from 18 to 69 years old, with different profiles, such as regions of birth (north, south, and southeast of Brazil) and educational levels. A web questionnaire was presented to the assessors with sentences about sentiment polarity and topic classification, in which each assessor rated 60 sentences, and each sentence was rated by at least 3 assessors. Sentiment polarity was classified into positive or negative, and the topic was classified into weak signals or other topics. In total, 3720 sentences were extracted from the OSN and evaluated by the assessors. For comparison, the same sentences were classified by the deep learning model, the CNN with BLSTM-RNN algorithm for classifying sentences with negative or positive polarity and topic classification.

The same sentences analyzed in the subjective tests were analyzed by the CNN with the BLSTM-RNN algorithm and by the sentiment analysis tool, the SentiMeter-Br and the LSTM with GRU model, in relation to the score obtained by the subjective tests. SentiMeter-Br was chosen because it can be easily updated for different scenarios, such as telecom services. Both metrics and models were trained for the Brazilian Portuguese language. In the case of SentiMeter-Br, specialists participated in updating its dictionary, where each word was analyzed by at least 3 specialists with the main words extracted from the Twitter OSN, totaling 107 new words for the telecom services context.

All these sentiment metrics were trained on the telecom scenario in the Brazilian Portuguese language. The SentiMeter-Br presents, as a result, a sentiment score varying from −5 to +5, and this score was converted to a positive or negative polarity. The results given by the LSTM with the GRU model did not need to be converted.

In the case of the LSTM with the GRU model, the LSTM layer used a 128-dimensional hidden state, a dropout of 0.5 fraction rate over the batch of sequences, and the LSTM layer was fed with a 128-dimensional hidden state that returns a single hidden state. Sigmoid activation was used according to the good results obtained in previous tests [36].

For the performance evaluation of the sentiment metrics, the % of assertiveness was used in relation to the sentiment polarity given by the assessors in subjective face-to-face tests.

### 3.3. Performance Measures

The classification accuracy, sensitivity or recall, precision, F-measure, G-mean, and area under the curve (AUC) were used to assess the effectiveness of each machine learning classifier. They were expressed as follows:

(4) $$Accuracy=\frac{TP+TN}{TP+TN+FP+FN}$$

(5) $$Sensitivity=\frac{TP}{TP+FN}$$

(6) $$Precision=\frac{TP}{TP+FP}$$

(7) $$\mathrm{F-measure}=\frac{2\times \left(precision\times recall\right)}{precision+recall}$$

(8) $$\mathrm{G-mean}=\sqrt{TP_{rate}\times TN_{rate}}$$

where TP, TN, FP and FN represent true positives, true negatives, false positives, and false negatives, respectively. $TP_{rate}=TP/p$ and $TN_{rate}=TN/n$, in which *p* represents the number of positive samples and *n* represents the number of negative samples.

The area under the curve (AUC) is given by (9).

(9) $$AUC=\int_{0}^{1}TP_{rate}\;\left(FP_{rate}^{-1}\left(t\right)\right)\;dt$$

where $FP_{rate}$ represents the false positive rate and *t* represents a varying parameter in range [0,1]. K-fold cross validation is performed in this work, in which 10-fold cross validation was used [96]; in this case, the sample was randomly partitioned into 10 subsamples of equal size. A subsample was used for validation, and the remaining nine subsamples were used as training data. Thus, 10-fold cross validation was used to obtain the accuracy of the polarity of the sentences and the topic of complaints.

## 4. Results and Discussion

This section describes the experimental results regarding the performance evaluation of the quality monitoring system.

### 4.1. Performance Evaluation of the CNN with BLSTM-RNN and SRS Activation Function in Relation to Other Algorithms

**Figure 4:**
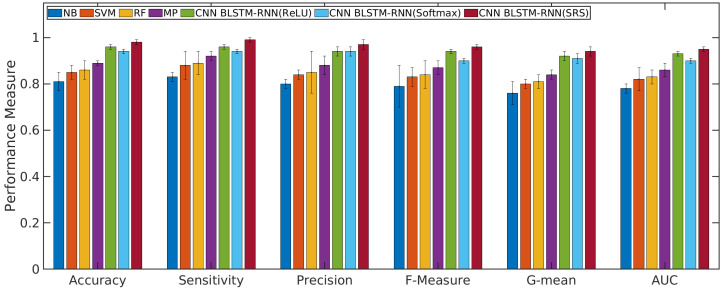
Results of machine learning algorithms for classifying the positive sentences.

**Figure 5:**
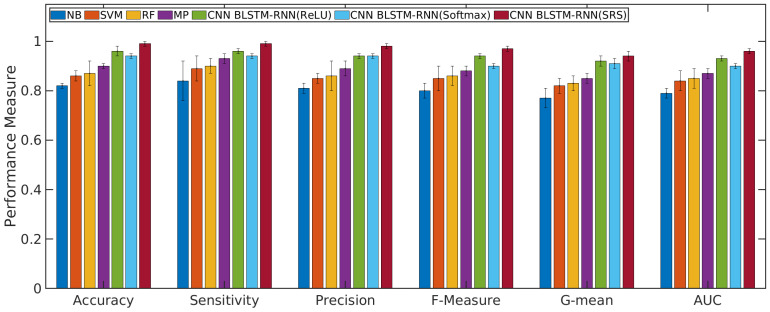
Results of machine learning algorithms for classifying the negative sentences.

According to Figure 4 and Figure 5, the CNN with BLSTM-RNN and SRS activation function achieved the highest average accuracy for positive/negative sentences with 0.98/0.99 ± 0.01, mean ± standard deviation, sensitivity of 0.99/0.99 ± 0.01, precision of 0.97 ± 0.02/0.98 ± 0.01, F-measure of 0.96/0.97 ± 0.01, G-mean of 0.93/0.94 ± 0.02, and AUC values of 0.95/0.96 ± 0.01 for classifying the positive/negative sentences. The CNN with BLSTM and other activation functions, such as softmax and ReLU, presented better results than the other algorithms. However, they did not achieve superior results to the SRS activation function.

In the tests, in the BLSTM-RNN algorithm, a batch size of 10, momentum of 0.8, learning rate of 0.01, 50 epochs and dropout rate of 0.5 were used. The values were chosen according to experimental tests.

**Figure 6:**
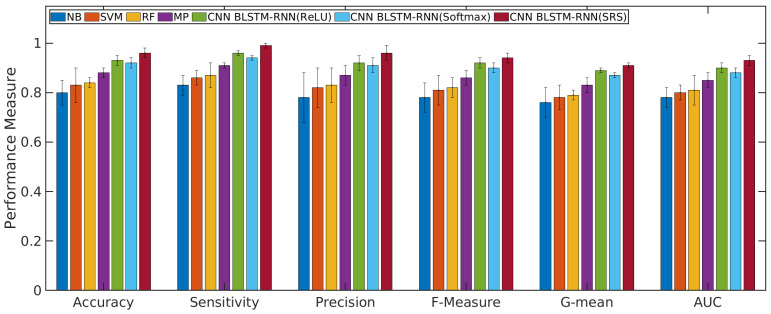
Results of machine learning algorithms to classify the topic of non weak signal.

**Figure 7:**
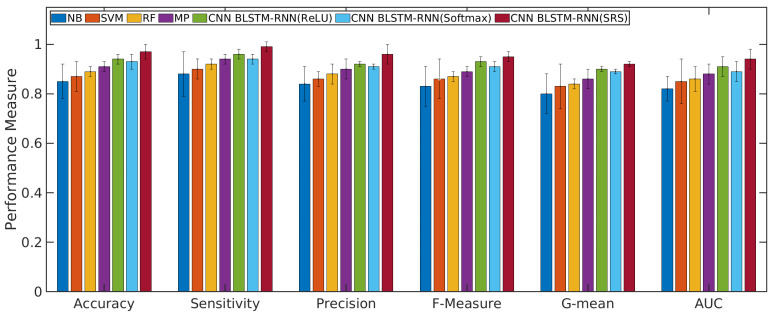
Results of machine learning algorithms to classify the topic of weak signal.

The results of machine learning algorithms in the validation phase for the complaint topics are presented in the following. Figure 6 presents the performance measure for other complaints or non weak signal complaint. Figure 7 presents the performance measure for a weak signal complaint. All Figs. show the average and standard deviation for each performance measure obtained from each classifier.

The time complexity of BLSTM-RNN presents superior values of training and execution time when compared to other machine learning algorithms. However, the time complexity of BLSTM-RNN with the SRS activation function presents similar values than other algorithms for the execution time and lower time complexity in comparison to the other CNN BLSTM-RNN algorithms, as presented in Table 1.

### 4.2. Detection of Social Network Complaints about Telecommunication Services and Signal Coverage of the RBS

Table 2 shows the results related to the detection of OSN complaints about telecom services. The operator names are represented by 1, 2, 3, and 4 for reasons of confidentiality. The results show that on average, 70.7% of complaints are related to a weak signal mainly caused by a lower number of RBSs. The other complaints are related to a large number of advertising messages, the costs of calls offered by the operators and whether a consumer experiences low throughput/frequent disconnections. This last complaint is related to a crowded area when the user experiences disconnections in his/her mobile phone, even if the user is very close to an RBS with a good signal.

As can be observed in Table 2, considering operator 4, the 79.1% of complaints in the OSN are related to the weak signal problem.

Table 3 shows the percentage values of the regions in which weak signal strength was verified, considering only the region in which there were users’ complaints about weak signals. The determination of regions with weak signals was determined using the ANATEL database. For example, considering operator 1, the 89% of all the regions that were indicated by users’ complaints had real weak signal or coverage problems, and the remaining 11% of the regions were not found to have the same technical problem. We can observe from Table 2 and Table 3 that the percentage of complaints regarding the weak signal has a high correlation with the real weak signal conditions, which are calculated using the ANATEL DB.

**Figure 8:**
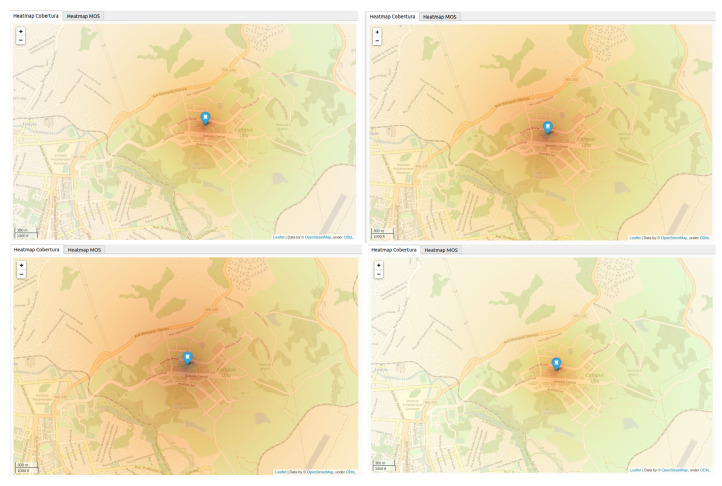
Signal strength results of an RBS located in MG state according to a developed software of operators 1, 2, 3, and 4, in which the map on the left of the first row represents operator 1, the map on the left in the second row represents operator 3. the map on the right of the first row represents operator 2, the map on the right in the second row represents operator 4.

In addition, for validation of the weak signal complaints, the coverage signal of RBSs was also visualized in a developed software using the Qt toolkit, Python and PyQt. Figure 8 presents the results of an RBS located in Minas Gerais state, considering the geographic region with respective buildings for each Brazilian network operator (1, 2, 3 and 4).

As an example of an RBS configuration data, Table 4 presents the data used in RBS of operator 1, whose signal intensity is presented in Figure 8.

Figure 8 shows the signal propagation, in which each point or pixel of the image represents the signal strength received in a geographical coordinates of the referent point. The areas with color in the strongest hue represent places with good signal coverage, and consequently, the areas with the lightest hue represent the places where the received signal strength is minimal or almost null.

### 4.3. Performance Evaluation of the Sentiment Model by Subjective Tests

The results of the subjective tests for evaluation of the deep learning model showed that of the sentences, 98% of the topics classified by the CNN with BLSTM-RNN were classified correctly.

Table 5 shows the results of the comparison of the CNN with BLSTM-RNN and SRS with other sentiment metrics/algorithms in relation to the subjective tests. As can be observed, the percentage of assertiveness of the sentiment polarity given by the CNN with BLSTM-RNN and SRS in relation to the subjective tests are higher than the results obtained by the SentiMeter-Br and the LSTM with GRU.

## 5. Conclusions

It is possible to observe that most of the complaints are related to weak signal strength after extracting the subscriber complaints on Twitter and the RBS. This work considered only this kind of complaint. However, other complaints can be extracted and used in applications in general for telecom services in next generation 5G or 6G-based networks, improving the users’ quality of experience (QoE). It is important to note that the proposed solution, Q-meter, can be used in future networks, as in the current networks, such as 3G and 4G.

The results presented in Figure 4, Figure 5, Figure 6 and Figure 7 show that the Q-Meter using sentiment analysis based on CNN with BLSTM-RNN with the SRS activation function obtained a precision of 0.97 for negative sentence classification and 0.96 for classifying the topic of weak signals. The CNN algorithm is used for extracting higher-level local features, and the BLSTM-RNN is responsible for extracting the bottleneck features and target-related feature representation. The solution shows that, through the deep learning model, it is possible to extract textual information.

Activation functions have a critical role in deep neural networks, and the use of the most effective activation function was explored. In this work, the SRS presented good results in relation to accuracy, sensitivity, precision, F-measure, G-mean, and AUC parameter. The time complexity using the SRS activation function was low compared to other algorithms because the SRS presents a better generalization performance as well as faster learning speed for model generation through batch normalization, accelerating deep network training. This work validates the use of the SRS activation function in the CNN with the BLSTM-RSS algorithm. The experiments showed significantly higher learning rates and proved the SRS properties as smoothness, nonmonotonicity, and boundedness. Additionally, the bounded property of the SRS activation function distinguishes itself from other activation functions.

The percentage of assertiveness of the CNN with BLSTM-RSS and the SRS presented higher values in relation to the other metrics, validating the proposal of using this algorithm solution for a telecom scenario.

The results regarding the main complaints in the OSN offered by the four selected companies showed that 79.1% of the complaints about weak signals in operator 4 were made by Twitter users, of which 92% had their veracity verified. Experimental results show that the number of complaints is highly related to real technical problems. Additionally, the proposed solution can analyze and detect other problems, such as commercial or marketing problems.

Currently, people use the OSN to express themselves, and this work concludes that it is possible to use this information medium for detecting problems and use these data for feeding applications or systems. The proposed Q-Meter is very useful in detecting specific regions with possible coverage problems effectively. This information should be very useful for cellular network operators, which can detect and solve problems, improving the user’s quality of experience. However, the proposed system, Q-Meter, presents some limitations, such as the identification of fake users complaints. In addition, in machine to machine network scenarios, if there is not a user opinion or complaint, then, the proposed solution cannot be applied in these networks.

## Figures and Tables

**Table 1:** Simulations time achieved by different methods and the proposed one for training and to execution time.

Method	Training	Execution
	(min)	(min)
NB	85	27
SVM	91	29
RF	101	31
MP	129	41
CNN BLSTM-RNN (ReLU)	157	51
CNN BLSTM-RNN (Softmax)	154	55
CNN BLSTM-RNN (SRS)	131	34

**Table 2:** Main complaints detection in the OSN about the telecommunications services offered by the four selected telecommunications companies.

Operator	Weak Signal	Other Complaint
1	66.0%	34.0%
2	67.0%	33.0%
3	70.7%	29.3%
4	79.1%	19.9%

**Table 3:** Percentage of OSN complaints that correspond to a weak signal found according to the ANATEL database.

Operator	Real Weak Signal
1	89%
2	91%
3	87%
4	92%

**Table 4:** Characteristics and values about the used RBS

Characteristics	Values
Environment	Conservative/City
RBS-Latitude	−19.9190677
RBS-Longitude	−43.9427914
Transmission Height	25.0 m
Transmission Power	6.0 W
Receiving Height	1.5 m
Receiving Gain	2.14 dBi
Initial Frequency	2670.00000000 MHz
Final Frequency	2690.00000000 MHz

**Table 5:** Results of comparison of the CNN with BLSTM-RNN and SRS with other metrics in relation to the subjective tests in percentage.

Sentiment Metric	% of Assertiveness
CNN with BLSTM-RNN and SRS	98%
SentiMeter-Br	89%
LSTM with GRU	92%

## Data Availability

Not applicable.
